# Oral Protein Supplements Might Improve Nutritional Status and Quality of Life in Elderly Patients after Standard Pancreatic Resection

**DOI:** 10.3390/nu16172988

**Published:** 2024-09-04

**Authors:** Na Rae Lee, Ho Kyoung Hwang, Hosun Lee, Chang Moo Kang

**Affiliations:** 1 Department of Nutrition Care, Severance Hospital, 50-1, Yonsei-Ro, Seodaemun-gu, Seoul 03722, Republic of Korea; naraelee@yuhs.ac; 2 Division of HBP Surgery, Department of Surgery, Yonsei University College of Medicine, 50-1, Yonsei-Ro, Seodaemun-gu, Seoul 03722, Republic of Korea; drhhk@yuhs.ac; 3 Pancreatobiliary Cancer Center, Yonsei Cancer Center, Severance Hospital, 50-1, Yonsei-Ro, Seodaemun-gu, Seoul 03722, Republic of Korea

**Keywords:** protein supplementation, nutritional status, quality of life (QoL), elderly post-surgical patients, pancreatic cancer, sarcopenia, muscle mass

## Abstract

Elderly patients who undergo pancreaticoduodenectomy (PPPD) or distal pancreatectomy (DP) experience not only a reduction in protein intake but also a decrease in protease secretion, leading to impaired protein digestion and absorption. This increases the risk of malnutrition and creates a dual burden of sarcopenia. This randomized, double-blind, placebo-controlled trial examined the impact of protein supplements on the nutritional status and quality of life (QoL) of elderly patients after PPPD and DP surgeries. For six weeks, the case group (CG; *n* = 23) consumed protein supplements containing 18 g of protein daily, while the placebo group (PG; *n* = 18) consumed a placebo with the same amount of carbohydrate. In elderly patients where protein digestion and intake were compromised, the CG showed significantly higher protein intake (77.3 ± 5.3 g vs. 56.7 ± 6.0 g, *p* = 0.049), improved QoL, better nutritional status, and faster walking speed compared to the PG. Protein intake was positively correlated with muscle mass and phase angle. Protein supplementation may not only increase protein intake but also improve clinical outcomes such as walking speed, nutritional status, and QoL in elderly post-surgical patients at high risk of sarcopenia. Further studies are needed to determine the optimal dosage and long-term effects.

## 1. Introduction

The pancreas plays a critical role in digestion by secreting enzymes that break down proteins, fats, and carbohydrates. Key proteases, including trypsin, chymotrypsin, and carboxypeptidase, are essential for digesting proteins into amino acids for absorption in the small intestine. Following pancreatic resection, there is a common reduction in the secretion of these enzymes, particularly in elderly patients over the age of 65, who are more likely to experience exocrine pancreatic insufficiency [1]. This decrease in enzyme production adversely affects patients’ food intake, quality of life, nutritional status, postoperative survival, and cancer-related outcomes [2].

A study conducted in Japan observed that patients undergoing pancreatic cancer surgery experienced significant declines in body weight, body fat, muscle mass, and quality of life over a three-month period, with a negative correlation between physical function and quality of life [3]. Similarly, a cross-sectional prospective study in Athens found that approximately 75% of pancreatic cancer patients had sarcopenia, and 80.3% were either at high risk of malnutrition or already malnourished [4].

Loss of muscle mass and strength occurs as aging progresses. Sarcopenia can be an additional burden for elderly patients suffering from underlying diseases [5]. In a meta-analysis study, it was reported that sarcopenia before surgery was highly prevalent in patients with digestive cancer. The study further reported that, as sarcopenia leads to an increase in the rate of postoperative morbidity and mortality, appropriate intervention before and after surgery is necessary [6]. In particular, studies have shown that sarcopenia has a negative impact after pancreatic surgery or during pancreatic cancer chemotherapy [7,8] and is related to the survival rate after biliary cancer surgery [9]. Hence, efforts need to be made to improve sarcopenia in pancreatobiliary diseases.

It is well established that muscle loss occurs due to an increase in muscle protein breakdown and a decrease in protein synthesis. Several studies have shown that, in cancer patients, muscle protein synthesis is not significantly impaired, indicating that additional protein intake can positively influence muscle protein synthesis [10]. However, in an observational study investigating the effects of nutritional support on the nutritional status of pancreatic cancer patients, it was found that, despite an increase in caloric intake post-surgery, protein intake and body weight significantly decreased [11].

The elderly population encounters a variety of complex nutritional challenges. These include a diminished sense of taste, reduced digestive capacity, and decreased appetite, all of which are common aspects of aging [12]. Such symptoms can lead to reduced nutrient intake, weight loss, and malnutrition. Importantly, malnutrition associated with aging is a risk factor that adversely affects patients’ clinical outcomes, quality of life, and physical functions [13]. Patients suffering from malnutrition exhibit higher morbidity and mortality than well-nourished patients and accrue greater medical expenses [14]. The prevalence of malnutrition among postoperative patients ranges from 35% to 60% [15], with even higher rates observed following pancreatic surgery, where the prevalence increases to 52% to 88% [16].

According to the “Nutritional Support and Hydration Guidelines for Elderly Patients” issued by the European Society for Clinical Nutrition and Metabolism (ESPEN) [17], oral nutrition supplements (ONSs) are advised for elderly patients who remain malnourished or at risk of malnutrition despite receiving nutritional counseling. The use of ONS can enhance nutrient intake and body weight, which helps decrease complications and hospital readmissions during hospitalization, and supports the maintenance of physical activity after discharge. In this regard, a nutritional intervention study on patients with head and neck cancer showed that the provision of ONS increased nutrient intake, mitigated weight loss, reduced the number of days spent in the hospital, decreased loss of appetite, and improved the patients’ QoL [18]. A prospective study involving pancreatic and biliary tract cancer patients undergoing chemotherapy demonstrated that nutritional status could be improved in the group receiving oral nutritional supplements (ONSs) by maintaining body composition [19].

Elderly patients over 65 who have undergone pancreatic resection often experience impaired protein digestion and absorption due to decreased secretion of digestive enzymes and are at higher risk of inadequate protein intake. This increases their risk of facing the dual burden of malnutrition and sarcopenia. However, well-designed randomized double-blind studies focusing on this population are scarce.

The purpose of this study is to explore the usefulness of protein supplements by administering protein supplement products to elderly patients aged 65 years or older who have undergone pancreatobiliary surgery and evaluating their quality of life, walking speed and skeletal muscle index (SMI) (indicators of sarcopenia), and nutritional status.

## 2. Materials and Methods

This study was a single-center, randomized, double-blind, and placebo-controlled clinical trial. It received approval from the Institutional Review Board (IRB) (project number: 4-2020-0552).

### 2.1. Research Subjects

The subjects of this study were elderly patients aged 65 years or older who have undergone surgery for pancreatobiliary cancer (pancreatic cancer, cholangiocarcinoma, intraductal papillary mucinous neoplasm, neuroendocrine tumor, and solid pseudopapillary tumor) between 7 January 2021 and 18 July 2022.

Patients were excluded from the study if they met any of the following criteria: (a) patients who were evaluated as being severely malnourished before surgery (PG-SGA grade C); (b) patients with an estimated glomerular filtration rate (eGFR) of less than 60 mL/min, or those diagnosed with chronic kidney disease, with a history of related treatment; (c) patients with a body mass index (BMI) of 30 kg/m^2^ or higher; (d) patients with ascites or edema severe enough to affect weight evaluation; (e) patients whose bioelectrical impedance analysis (BIA) cannot be measured due to the use of pacemakers or implants; (f) patients who were evaluated by the researcher in charge as being unable to participate in the study for psychological or cognitive reasons; and (g) patients who did not consent to this study.

This study aimed to achieve 80% statistical power while maintaining a significance level of 5%. The sample size calculation was based on the Korean Dietary Reference Intakes (DRI) for adults aged 65 and older. The protein requirement to maintain nitrogen balance in this population, set at 0.73 g/kg, was assumed to be the population mean for Group 1. Since postoperative patients are recommended to consume at least 1.5 g/kg of protein, this value was assumed as the population mean for Group 2. Based on these assumptions, the calculated sample size was 27 participants per group. To account for an anticipated dropout rate of approximately 10%, the final sample size was adjusted to 30 participants per group.

**Figure 1:**
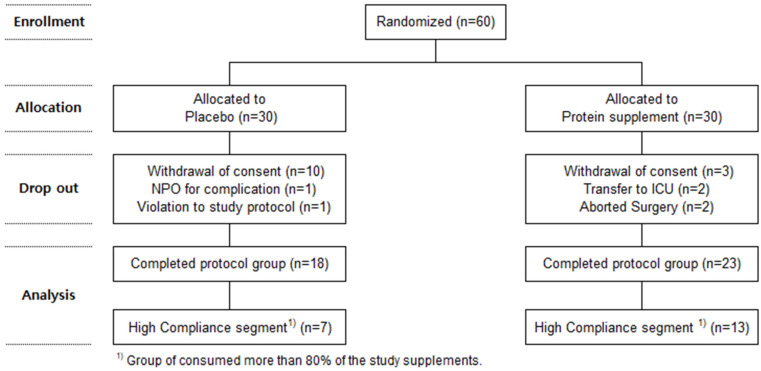
Flowchart of the study.

Sixty patients who agreed to participate in the study were registered as research subjects. The registered subjects were randomly assigned to the CG (Case Group) and the PG (Placebo Group) using a random number table. Thirteen subjects withdrew their consent during the study. Additionally, one patient who required fasting due to complications, one patient who did not adhere to the study protocol, two patients who were transferred to the ICU, and two patients whose surgeries were discontinued were classified as dropouts. Data from 41 patients who completed the final study protocol were analyzed. It was deemed that patients who consumed significant amounts of supplements could more effectively demonstrate the impact of varying protein intakes. Therefore, 20 patients who consumed more than 80% of the research product were defined as a high-compliance segment, and further analysis was conducted on this group (Figure 1).

### 2.2. Research Method

The study subjects were asked to take supplements every day for a period of 42 days (six weeks) from the date on which they commenced oral intake thereof after surgery. Patients in the CG were provided with 140 kcal, 18 g of protein, and 13 g of carbohydrate each day through protein supplements, while patients in the PG were given 140 kcal, 0 g of protein, and 31 g of carbohydrate through placebo supplements containing the same amount of carbohydrate in lieu of protein (Supplementary Material). The packages of the supplements used in this study were identical and were managed by a third party who assigned specific numbers to the packages so that the researchers and subjects could not distinguish them.

### 2.3. Methods of Data Collection and Evaluation

The points in time of data collection were as follows: day before surgery (pre-OP), within 24 h from the determined date of discharge (DC), and at the time of the first outpatient visit after the surgery (OPD). Electronic medical records were used to collect data on the subjects’ general characteristics such as their diagnosis, gender, age, height and weight measured at the time of hospitalization, and BMI. A trained clinical dietitian evaluated the subjects’ nutritional status and intake as well as muscle mass and walking speed, which are indicators related to sarcopenia, and conducted a survey to evaluate the patients’ quality of life.

#### 2.3.1. Evaluation of Nutritional Status

The Patient-Generated Subjective Global Assessment (PG-SGA) was utilized to evaluate the nutritional status of cancer patients. Patients independently recorded changes in their weight, dietary intake, symptoms impacting their intake, and activity level. The clinical dietitian assessed each patient’s diagnosis, degree of metabolic stress, loss of body fat and muscle, and degree of edema. This assessment was integrated with the patient’s independent records, and the nutritional status of each patient was then evaluated as “good (PG-SGA grade A)”, “moderate malnutrition (PG-SGA grade B)”, or “severe malnutrition (PG-SGA grade C)”. Nutritional management varied based on the total score: scores of 0 to 1 indicated no intervention was necessary; scores of 2 to 3 required nutritional counseling for the patient and their family; scores of 4 to 8 necessitated nutritional management and intervention by a clinical dietitian; and scores of 9 or higher warranted intensive nutritional intervention by a clinical dietitian.

#### 2.3.2. Evaluation of Intake

Usual intake before surgery was investigated using the 24 h recall method. During the hospitalization period, the patients’ daily intake was recorded using the self-developed “meal intake during hospitalization” survey. The patients were asked to write a “meal journal” and bring it with them at the time of their first outpatient visit. The patients’ nutrient intake was analyzed using the “CAN-Pro 5.0 (Web ver.) (Korean Nutrition Society, Seoul, Korea)” program. The energy requirements of the study subjects were calculated by multiplying the Resting Energy Expenditure (REE), computed using the Mifflin-St. Jeor Equation, by 1.2 or 1.3. The protein requirements were calculated by multiplying the unit weight by 1.2 or 1.3, and energy and protein requirements were adjusted according to the patients’ conditions.

#### 2.3.3. Evaluation of Quality of Life

The patients’ quality of life was assessed using the “European Organization for Research and Treatment in Cancer 30 (EORTC QLQ-C30)” questionnaire, which is a questionnaire designed to evaluate the quality of life of cancer patients. The final score was calculated by adding the scores for the last two questions out of 30 evaluation questions on the subjects’ overall quality of life. The higher the quality of life (QoL) score, the better the quality of life.

#### 2.3.4. Evaluation of Indicators Related to Sarcopenia

According to the studies conducted by the European Working Group on Sarcopenia in Older People (EWGSOP2; 2019) [20] or the Asian Working Group for Sarcopenia (AWGS; 2019) [21] regarding the definition and diagnosis of sarcopenia, sarcopenia is defined by using a combination of muscle mass assessment, muscle strength measurement, and physical activity assessment. In this study, muscle mass evaluation and physical activity assessment were conducted.

- Evaluation of Walking Speed

Walking speed was assessed by measuring the time taken to walk 10 m on a flat, straight surface. For inpatients, the 10 m distance was clearly marked on the ward floor, and for outpatients, it was marked in the clinic hallway. Before starting the measurement, participants stood still at the starting line. The researcher instructed participants to walk at their usual pace. The time taken to walk the 10 m was recorded with a stopwatch from the moment the participant began walking. The measurement was repeated three times, and the average of these three trials was used for analysis.

- Evaluation of Muscle Mass

Muscle mass was measured three times (before surgery, upon discharge, and at the time of the first outpatient visit) using “InBody S10 (InBody^®^, Seoul, Korea)” featuring the bioelectrical impedance analysis (BIA) method. Body cell mass (BMC), PA, and SMI were utilized for the analysis. SMI was calculated by dividing the sum of the muscle mass of the limbs excluding the torso (appendicular skeletal muscle mass; ASM) by the square of the height converted to meters [SMI = ASM/height^2^].

### 2.4. Method of Statistical Analysis

Statistical analysis was conducted using the Statistical Analysis Systems Package Version 9.4 (SAS Institute, Cary, NC, USA). All data used for the statistical analysis were analyzed on a per-protocol (PP) basis, excluding the data corresponding to those who dropped out. Any data exceeding IQR × 1.5 were removed prior to the analysis.

For the purpose of comparison between the two groups at each time point, continuous variables were analyzed using Student’s *t*-test and categorical variables were analyzed using the chi-square test. Changes in each group at the time of each of the three visits were compared by using the Linear Mixed-Effects Model (LMM).

Additionally, Spearman correlation analysis was performed to evaluate the correlation between the protein intake and muscle mass in the high-compliance segment.

## 3. Results

Among the subjects, 18 had been diagnosed with pancreatic cancer, 14 with bile duct cancer, and 9 with other cancers. Thirty-one subjects had undergone pylorus-preserving pancreaticoduodenectomy (PPPD) and 10 patients had undergone distal pancreatectomy (DP). There were no statistically significant differences in terms of gender, age, height, weight, BMI, or PG-SGA between the two groups. No significant differences were found in the baseline characteristics of the two groups in the high-compliance segment, either (Table 1).

### 3.1. Evaluation of Nutritional Status (Figure 2)

**Figure 2:**
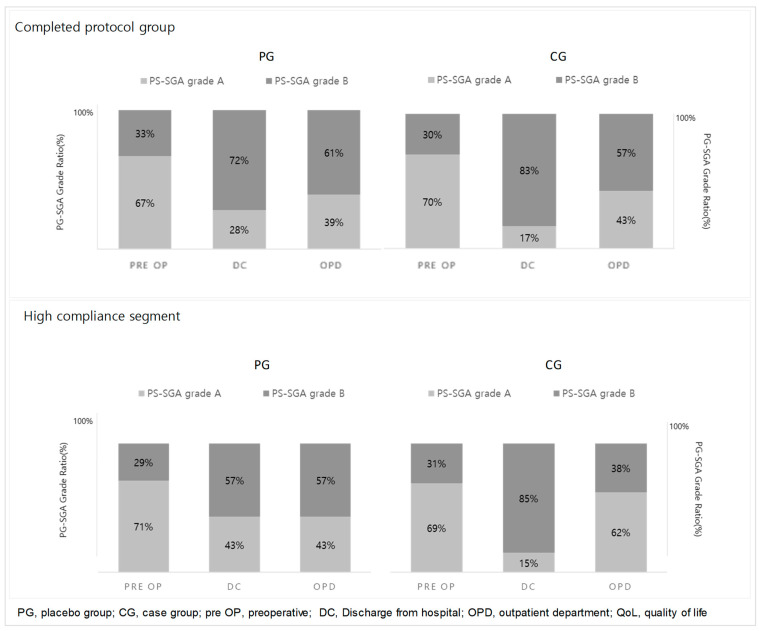
Comparison of PG-SGA grades between the PG and CG.

The patients’ nutritional status (PG-SGA grade) at each time point of their visits is presented in Figure 2. In the CG, 16 patients (70%) had good nutritional status before surgery and 7 patients (30%) had moderate malnutrition. Meanwhile, there were 12 patients (67%) in the PG with good nutritional status and 6 patients (33%) with moderate malnutrition. At the time of discharge, 4 patients (17%) in the CG and 5 patients (28%) in the PG were in good nutritional status, whereas 19 patients (83%) in the CG and 13 patients (72%) in the PG were evaluated as moderately malnourished. The proportion of moderately malnourished patients increased in both groups at the time of discharge and at the time of outpatient visits compared to pre-surgery, but there was no statistically significant difference between the two groups.

In both the CG and the PG, the PG-SGA score increased at each time point compared to pre-surgery, but there was no statistically significant difference between the two groups. At the time of discharge (DC), the PG-SGA score was nine or higher for both the PG (9.7 ± 0.9) and the CG (9.6 ± 0.8), indicating that intensive nutritional management and intervention by a clinical dietitian was recommended to improve the symptoms.

In both the PG and the CG of the high-compliance segment, the rate of moderate malnutrition (PG-SGA grade B) increased at the time of discharge and at the time of the first outpatient visit compared to pre-surgery. In the PG, there was no difference in the proportion of patients with moderate malnutrition (PG-SGA grade B) at the time of discharge and at the time of the first outpatient visit. In the CG, whereas the proportion of patients with moderate malnutrition (PG-SGA grade B) at the time of discharge was 85% (11 patients), it decreased by approximately 47% (six patients) to 38% (five patients) at the time of the outpatient visit. However, there was no statistically significant difference.

### 3.2. Analysis and Comparison of Nutrient Intake (Table 2)

The rate of supplement intake was 73.8 ± 4.0%, and there was no difference in the supplement intake rate between the PG and the CG (75.1 ± 4.0% vs. 72.9 ± 6.1%, *p* = 0.824). The supplement intake rate of the high-compliance segment was 94.0 ± 1.4%. Again, there was no difference in the supplement intake rate between the PG and the CG (94.3 ± 1.9 vs. 93.9 ± 1.9, *p* = 0.888). After discharge, protein intake in the CG was statistically significantly greater than in the PG (77.29 ± 5.34 g per day vs. 56.66 ± 6.03 g per day, *p* = 0.049). In the high-compliance segment, protein intake was greater in the CG than in the PG, but the difference was not statistically significant. In both the group that completed the protocol and the high-compliance segment, the energy and fat intake of the CG was higher than that of the PG, but the difference was not statistically significant.

**Table 2:** Comparison of nutrition intake between two groups.

Variables ^(1)^		Completed Protocol Group							High Compliance Segment						
	Visit	PG (*n* = 18)			CG (*n* = 23)			*p*-Value ^(2)^	PG (*n* = 7)			CG (*n* = 13)			*p*-Value ^(2)^
Energy (kcal/day)	pre OP	1549.3	±	99.4	1678.2	±	88.0		1481.4	±	166.4	1811.7	±	122.1	
	DC	770.6	±	99.4	879.2	±	88.0	0.699	917.4	±	166.4	1024.1	±	122.1	0.276
	OPD	1406.8	±	99.4	1530.9	±	88.0	0.819	1566.6	±	166.4	1735.9	±	122.1	0.705
Carbohydrate (g/day)	pre OP	206.2	±	12.6	224.0	±	11.1		190.0	±	17.5	243.3	±	12.8	
	DC	90.9	±	12.6	88.4	±	11.1	0.319	99.2	±	17.5	99.8	±	12.8	0.077
	OPD	209.6	±	12.6	214.1	±	11.1	0.514	225.4	±	17.5	249.6	±	12.8	0.322
Protein (g/day)	pre OP	72.9	±	6.0	77.3	±	5.3		70.7	±	10.2	85.0	±	7.5	
	DC	34.1	±	6.0	52.1	±	5.3	0.090	45.1	±	10.2	61.9	±	7.5	0.629
	OPD	56.7	±	6.0	77.3	±	5.3	0.049	67.6	±	10.2	87.9	±	7.5	0.475
Fat (g/day)	pre OP	48.9	±	4.6	52.4	±	4.1		49.1	±	8.0	56.5	±	5.8	
	DC	29.3	±	4.6	34.6	±	4.1	0.968	37.0	±	8.0	41.0	±	5.8	0.658
	OPD	37.3	±	4.6	42.9	±	4.1	0.973	43.9	±	8.0	48.3	±	5.8	0.548

PG, placebo group; CG, case group; pre OP, preoperative; DC, Discharge from hospital; OPD, outpatient department; ^(1)^ LSmean ± SE (all such values). ^(2)^ Linear mixed-effect model was used to analyze the group and time interaction for CG compared with PG during study period.

### 3.3. Quality of Life (Table 3)

The quality of life (QoL) score of the CG at the time of discharge (DC) (8.68 ± 0.52) was significantly higher than that of the PG (7.40 ± 0.60) (*p* = 0.016). The QoL score of the CG at the time of the first outpatient visit (1st OPD) (9.09 ± 0.47) was also statistically significantly higher than that of the PG (8.33 ± 0.54) (*p* = 0.042). As for the QoL score of the high-compliance segment, there was no difference in the QoL scores of the CG and the PG before surgery and at the time of discharge. However, at the time of the first outpatient visit, the QoL score of the CG was statistically significantly higher than that of the PG (10.0 ± 0.63 vs. 7.86 ± 0.86, *p* = 0.017).

**Table 3:** Comparison of QoL, walking speed, and body composition between two groups.

Variables ^(1)^		Completed Protocol Group							High Compliance Segment						
	Visit	PG (*n* = 18)			CG (*n* = 23)			*p*-Value ^(2)^	PG (*n* = 7)			CG (*n* = 13)			*p*-Value ^(2)^
QoL (score)	pre OP	9.8	±	0.6	9.4	±	0.5		9.8	±	1.0	9.6	±	0.7	
	DC	7.4	±	0.6	8.7	±	0.5	0.016	7.8	±	0.9	9.1	±	0.7	0.066
	OPD	8.3	±	0.5	9.1	±	0.5	0.042	7.9	±	0.9	10.0	±	0.6	0.017
10-m walking speed (s)	pre OP	10.5	±	0.6	9.7	±	0.6		9.9	±	1.0	9.5	±	0.7	
	DC	11.6	±	0.6	10.4	±	0.6	0.240	11.9	±	1.0	10.2	±	0.7	0.035
	1st OPD	9.2	±	0.6	9.4	±	0.6	0.628	8.8	±	1.0	9.7	±	0.7	0.651
Body Composition															
BCM (kg)	pre OP	30.7	±	1.4	32.5	±	1.3		29.4	±	2.3	33.6	±	1.7	
	DC	31.2	±	1.4	32.4	±	1.3	0.446	29.7	±	2.3	33.4	±	1.7	0.738
	1st OPD	29.7	±	1.4	31.5	±	1.3	0.928	28.7	±	2.3	33.1	±	1.7	0.891
PA (°)	pre OP	5.5	±	0.2	6.1	±	0.2		5.6	±	0.4	6.5	±	0.3	
	DC	5.3	±	0.2	5.8	±	0.2	0.747	5.3	±	0.4	6.0	±	0.3	0.667
	1st OPD	4.7	±	0.2	5.5	±	0.2	0.365	4.8	±	0.4	5.7	±	0.3	0.999
ASM (kg)	pre OP	20.0	±	1.2	21.3	±	1.1		18.9	±	1.9	22.0	±	1.4	
	DC	20.5	±	1.2	21.3	±	1.1	0.413	19.1	±	1.9	22.0	±	1.4	0.875
	1st OPD	20.2	±	1.2	21.5	±	1.0	0.823	19.1	±	1.9	22.7	±	1.4	0.578
SMI (kg/m^2^)	pre OP	7.6	±	0.3	8.0	±	0.2		7.6	±	0.5	8.1	±	0.3	
	DC	7.8	±	0.3	8.0	±	0.2	0.249	7.7	±	0.5	8.1	±	0.3	0.715
	1st OPD	7.7	±	0.3	8.1	±	0.2	0.615	7.7	±	0.5	8.4	±	0.3	0.700

PG, placebo group; CG, case group; pre OP, preoperative; DC, Discharge from hospital; OPD, outpatient department; QoL, quality of life; BCM, Body Cell Mass; PA, Phase Angle; ASM, Appendicular Skeletal Muscle Mass; SMI, Skeletal Muscle Mass Index. ^(1)^ LSmean ± SE (all such values). ^(2)^ Linear mixed-effect model was used to analyze the group and time interaction for CG compared with PG during study period.

### 3.4. Evaluation of 10 m Walking Speed (Table 3)

In both the CG and the PG, walking speed increased at the time of discharge compared to pre-surgery. The walking speed became faster at the time of the first outpatient visit compared to the time of discharge, but there was no statistically significant difference. The walking speed of the CG of the high-compliance segment was significantly faster than that of the PG at the time of discharge (10.17 ± 0.73 s vs. 11.92 ± 0.97 s, *p* = 0.035).

### 3.5. Evaluation of Muscle Mass

There was no difference between the PG and the CG in terms of the BMC, PA, ASM, and SMI, measured using the bioelectrical impedance analysis (BIA) method. (Table 3) Meanwhile, to evaluate the interaction between protein intake and muscle mass, Spearman correlation analysis was performed only on the high-compliance segment. The PA and the SMI at the time of the first outpatient visit had a positive correlation with the total protein intake during hospitalization (total protein intake vs. PA, |R| = 0.470, *p* = 0.002, total protein intake vs. SMI, |R| = 0.427, *p* during hospitalization. = 0.005) and the total protein intake after discharge (total protein intake vs. PA, |R| =0.55, *p* < 0.0001, total protein intake vs. SMI, |R| = 0.37, *p* = 0.019) (Table 4).

## 4. Discussion

Our study provides additional data supporting previous research that emphasizes the increase in sarcopenia and nutritional imbalances following pancreatic cancer and pancreatic resection. In particular, elderly patients who undergo pancreaticoduodenectomy (PPPD) or distal pancreatectomy (DP) may experience not only reduced protein intake but also decreased protease secretion, leading to impaired protein digestion and absorption. These factors further increase the risk of sarcopenia in elderly patients, potentially hindering post-surgical recovery. This study empirically confirms these issues, suggesting that protein supplementation may improve the nutritional status and physical function of this patient population.

Although there was no significant difference in energy intake between the two groups in our study, protein intake in the CG group was statistically significantly higher at discharge (*p* = 0.049). This outcome is consistent with the findings of a study conducted in Korea, which discovered that the group that consumed more than 1.2 g/kg of protein suffered less sarcopenia compared with the group that consumed less than 0.8 g/kg of protein [22], or the meta-analysis which suggested that inadequate protein intake may be related to sarcopenia in the elderly [23]. Protein intake at the time of the CG’s first outpatient visit was 1.37 ± 0.09 g, which was lower than the intake in the study where muscle loss was improved in the group that consumed 1.5 g/kg of protein for 12 weeks [24] or the protein intake recommended by the expert group of the ESPEN; this suggested that critically ill patients or elderly patients with severe trauma take 1.6 g/kg of protein to prevent sarcopenia. However, in the CG of the high-compliance segment, protein intake at the time of the first outpatient visit was 1.51 ± 0.13 g per body weight (1 kg), aligning closely with the results from the DSM Ten Haaf study [24] and the intake recommended by the ESPEN.

In this study, the subjects were instructed to take protein supplements for a period of 42 days (six weeks), but there was a gap in the timelines of research conducted domestically and internationally. A majority of the studies spanned 12 to 13 weeks [25,26,27]. Some studies involved interventions in which the subjects were asked to consume ONS for a period of 6 to 12 months, which resulted in fat loss, muscle mass gain, and improved nutritional status [28,29,30]. Considering the findings of a meta-analysis study that high protein intake does not prevent sarcopenia or the decline in physical functions over time [31], further clinical research is necessary to determine the optimal protein intake and duration, taking age and health conditions into account.

In addition, according to the recommendations from the expert group of the ESPEN [32], as the decline in muscle mass due to aging usually results from a decrease in muscle protein synthesis rather than an increase in muscle protein breakdown, daily physical activity or resistance exercise is recommended in addition to sufficient protein intake. Additionally, some studies have indicated that muscle mass was improved when workout was combined with long-term intake of protein supplement products [28,29]. In the future, studies need to be conducted by mediating not only the intake of protein supplement products but also muscle strength training by taking the characteristics of the study subjects into consideration.

Authors should discuss the results and how they can be interpreted from the perspective of previous studies and of the working hypotheses. The findings and their implications should be discussed in the broadest context possible. Future research directions may also be highlighted.

Numerous studies have suggested that both the quality of life and nutritional status of patients deteriorate within three months following pancreatectomy surgery [33,34]. Consistently, in this study, all indicators related to quality of life and nutritional status showed a decline at each measured point post-surgery. This aligns with findings from a retrospective study indicating an increased risk of malnutrition post-surgery, with nutritional indicators deteriorating rapidly within about three months after pancreatectomy [35]. Another study demonstrated that nutritional status improved in patients who consistently consumed protein supplements [36]. In this context, the current study found that the rate of moderate malnutrition in the CG of the high-compliance segment decreased from 85% to 38%. These results underscore that patients’ nutritional status is likely to improve and their recovery period is also likely to be shortened if they faithfully consume their protein supplements.

In this study, the quality of life (QoL) scores of the CG were higher than those of the PG at the time of discharge (7.4 ± 0.6 vs. 8.7 ± 0.5, *p* = 0.016) and at the time of the first outpatient visit (9.1 ± 0.5 vs. 8.3 ± 0.5, *p* = 0.042). Considering the outcome of the study which found that weight loss and malnutrition after abdominal digestive surgery lead to a decline in the quality of life [37] or the study which found that, in elderly people, insufficient protein intake limits their mobility and worsens their physical function and quality of life [38,39], it is deemed that the improvement in nutritional status resulting from increased protein intake has contributed to the improvement in the QoL scores in this study. Moreover, in this study, at the time of the first outpatient visit, the CG (5.50 ± 0.19) had a greater PA than the PG (4.74 ± 0.21), and the PA of the CG of the high-compliance segment (5.72 ± 0.26) was also greater than that of the PG (4.8 ± 0.21) and was 5.0 or larger. Meanwhile, PA has a close correlation with the prognosis and mortality of cancer patients. The worse a patient’s nutritional status, the smaller the PA. The findings of this study can be regarded as a meaningful outcome, considering the study which showed that the survival period of patients with pancreatic cancer declines when their PA is less than 5.0 [40,41].

There have been studies in which the compliance rate for product intake was as low as 50% to 65%, depending on the period of the study and the study subjects [28,30]. However, in general, the compliance rate for product intake was 75% to 80% or higher in most studies [25,26,27,42]. The compliance rate for product intake in this study was 73.8 ± 1.0%. In the high-compliance segment, the QoL score (7.9 ± 0.9 vs. 10.0 ± 0.6, *p* = 0.017) and the 10 m walking speed (11.9 ± 1.0 vs. 10.2 ± 0.7, *p* = 0.035) showed statistically significant differences in the CG compared to the PG. This indicates that it is important not only to increase the supply of protein but also to enhance intake compliance. Through a meta-analysis study, S Onvani et al. reported that individualized consultation provided by a clinical dietitian can help to improve the energy level and the protein intake of the elderly who are at high nutritional risk [43]. In its “Nutritional Support and Hydration Guidelines for Elderly Patients [17]”, the ESPEN recommended improving nutrient intake primarily through nutrition counseling. Therefore, integrating protein supplements into clinical practice could be enhanced by promoting active consumption through personalized counseling provided by a clinical dietitian.

This short-term study spanning six weeks aimed to assess the effects of protein supplement intake before and after surgery, but it did not evaluate long-term recovery outcomes such as mortality rates, complication rates, or the duration of chemotherapy. It is anticipated that these aspects could be assessed through retrospective research on the subjects of this study.

The dropout rate in this study was 31.7%, which is higher than that observed in other studies. Such a high dropout rate is attributed to the advanced age (over 65 years) of the study subjects and the high severity of pancreatobiliary cancer. Future studies focusing on severe diseases in elderly populations should consider such high dropout rates when planning the number of subjects.

## 5. Conclusions

Administering protein supplements for a period of 42 days (6 weeks) to elderly patients over 65 years who underwent standard pancreatic resection resulted in increased protein intake. A notable finding was that higher compliance with supplement intake correlated with improved quality of life (QoL) scores and quicker walking speeds. Additionally, a positive relationship was observed where increases in total protein intake were associated with rises in the SMI and the PA, suggesting that adequate oral intake of protein supplements enhances helps to improve patients’ quality of life and nutritional status while alleviating sarcopenia.

Further research involving different types of cancer patients, taking factors such as protein intake, duration of intake, and exercise interventions into account, could be valuable in enhancing the nutritional status, muscle mass, and quality of life among elderly patients with cancer.

## Figures and Tables

**Table 1:** Baseline characteristics of the subjects.

Variables ^(1)^	Completed Protocol Group										High Compliance Segment									
	All (*n* = 41)			Placebo (*n* = 18)			Protein Supplement (*n* = 23)			*p*-Value ^(2)^	All (*n* = 20)			Placebo (*n* = 7)			Protein Supplement (*n* = 13)			*p*-Value ^(2)^
Gender (male/female)	21/20			11/7			10/13			0.767	7/13			4/3			9/4			1.000
Age (yr)	72.6	±	0.8	73.2	±	1.3	72.1	±	1.0	0.496	73.7	±	1.1	75.0	±	1.7	73.0	±	1.4	0.380
Ht (cm)	161.4	±	1.5	161.3	±	1.8	161.5	±	2.2	0.810	161.2	±	2.2	157.4	±	2.4	163.3	±	3.1	0.218
Wt (kg)	60.6	±	1.6	59.7	±	2.0	61.4	±	2.3	0.987	61.8	±	2.5	57.8	±	2.8	63.9	±	3.4	0.249
BMI (kg/m^2^)	23.2	±	0.4	23.0	±	0.7	23.4	±	0.5	0.923	23.6	±	0.6	23.3	±	1.1	23.8	±	0.8	0.732
Diagnosis										0.437										1.000
Pancreatic cancer	18			10			8				8			4			4			
Cholangiocacinoma	14			6			8				7			3			4			
Others	9			2			7				5			0			5			
Operation (PPPD/DP)	31/10			14/4			17/6			0.703	14/6			5/2			9/4			1.000
PG-SGA																				
Grade A/B/C	28/13/0			12/6/0			16/7/0			0.843	14/6/0			5/2/0			9/4/0			1.000
Score	5.3	±	0.5	5.1	±	0.7	5.6	±	0.7	0.971	4.8	±	0.7	4.7	±	1.0	4.8	±	0.9	0.930

^(1)^ Data are the means ± standard error: Age, Ht, Wt, BMI, PG-SGA score. ^(2)^ Student *t*-test for continuous variables and Chi-square test for categorical variables were used to compare the difference between the groups.

**Table 4:** Correlation between protein intake and body composition.

Variable			PA		SMI	
			DC	OPD	DC	OPD
Total Oral Protein	DC	|R| ^(1)^	0.29	0.47	−0.02	0.43
		*p*-Value	0.060	0.002	0.877	0.005
	OPD	|R|	0.27	0.55	0.09	0.37
		*p*-Value	0.082	0.000	0.561	0.019

PA, Phase Angle; SMI, Skeletal Muscle Mass Index; DC, Discharge from hospital; OPD, outpatient department. ^(1)^ 0 < |R| < 0.45: low correlation, 0.45 < |R| < 0.75: moderately correlation, 0.75 < |R|: strong correlation.

## Data Availability

The data supporting the reported results can be found at ClinicalTrials.gov, identifier NCT06570174.

## Supplementary Materials

The following supporting information can be downloaded at: https://www.mdpi.com/article/10.3390/nu16172988/s1, Table S1: Nutrition facts of study material.
