# Supplementary material for: Oral Protein Supplements Might Improve Nutritional Status and Quality of Life in Elderly Patients after Standard Pancreatic Resection
Source: Nutrients. 2024 Sep 4;16(17):2988. doi: 10.3390/nu16172988 (PMC11397451; doi:10.3390/nu16172988)
Supplement: Supplementary file 1 [file nutrients-16-02988-s001.zip › nutrients-3173695-supplementary.pdf]

Table S1. Nutrition facts of study material.

	Protein supplement	Placebo supplement
Energy (kcal)	140	140
Carbohydrate (g)	13	31
Protein (g)	18	0
Fat (g)	1.5	1.5
Sodium (g)	310	310
Fiber (g)	-	2
Fructo-oligosaccharides (g)	4	4
Vitamin B_6 (mg)	3	3
Vitamin D (mg)	20	20
Calcium (mg)	304	304
Magnesium (mg)	100.8	100.8
Zinc (mg)	2.55	2.55
